# Running Online Behavioral Experiments Using R: Implementation of a Response-Time Decision Making Task as an R-Shiny App

**DOI:** 10.5334/joc.200

**Published:** 2022-01-07

**Authors:** Agustín Perez Santangelo, Guillermo Solovey

**Affiliations:** 1 Instituto de Investigación en Ciencias de la Computación, Universidad de Buenos Aires, (UBA), Consejo Nacional de Investigaciones Científicas y Técnicas (CONICET), AR; 2 Laboratorio de Neurociencia, CONICET, Universidad Torcuato Di Tella, AR; 3 Instituto de Cálculo, Facultad de Ciencias Exactas y Naturales, UBA-CONICET, AR

**Keywords:** online experiments, R-Shiny, response time, numerical cognition

## Abstract

Online experiments allow for fast, massive, cost-efficient data collection. However, uncontrolled conditions in online experiments can be problematic, particularly when inferences hinge on response-times (RTs) in the millisecond range. To address this challenge, we developed a mobile-friendly open-source application using R-Shiny, a popular R package. In particular, we aimed to replicate the numerical distance effect, a well-established cognitive phenomenon. In the task, 169 participants (109 with a mobile device, 60 on a desktop computer) completed 116 trials displaying two-digit target numbers and decided whether they were larger or smaller than a fixed standard number. Sessions lasted ~7-minutes. Using generalized linear mixed models estimated with Bayesian inference methods, we observed a numerical distance effect: RTs decreased with the logarithm of the absolute difference between the target and the standard. Our results support the use of R-Shiny for RT-data collection. Furthermore, our method allowed us to measure systematic shifts in recorded RTs related to different OSs, web browsers, and devices, with mobile devices inducing longer shifts than desktop devices. Our work shows that precise RT measures can be reliably obtained online across mobile and desktop devices. It further paves the ground for the design of simple experimental tasks using R, a widely popular programming framework among cognitive scientists.

## Introduction

Online experiments are gaining ground among behavioral and cognitive science researchers as they allow collecting massive data from diverse participants remotely, quickly, and at a low cost. Moreover, they became the principal data collection tool during the COVID-19 pandemic to keep projects running. A particularly valuable source of information in experimental psychology is participants’ response times (RTs). While RTs are crucial to infer many psychological processes (e.g. Stroop interference effect), RT measures are extremely susceptible to noise and interference. Oftentimes, millisecond-range precision is required to detect effects on RT (Plant, 2016). Thus, assessing the reliability of RT data obtained in web-based experiments is paramount.

To illustrate what an online setting implies, picture yourself riding a bus on your way back home when a message pops up on your phone inviting you to participate in a decision-making task. The bus rocks constantly, it is noisy and you are somewhat tired, but decide to partake anyway. After all, you are only asked to make a handful of simple and quick decisions in 5 minutes. Though this may be an extreme example, it shows how uncontrolled conditions in online studies contrast sharply with standard laboratory experiments. In the latter, participants are welcomed by a researcher, receive detailed instructions, and complete hundreds of trials during several minutes in front of a calibrated computer in a quiet room.

Online behavioral experiments are not novel, as attempts to use the web as a tool for psychological research date back to the late 1990s when the internet became more accessible (Krantz & Dalal, 2000; Reips, 2002). The reliability of data collected online has always been a focus of attention (Anwyl-Irvine et al., 2020; Brown et al., 2014; Crump et al., 2013; Dandurand et al., 2008; Dufau et al., 2011; Garaizar & Reips, 2019; Germine et al., 2012; Gosling et al., 2004; Grootswagers, 2020; Huber et al., 2019; Kochari, 2019; Krantz & Dalal, 2000; Pronk et al., 2020; Semmelmann & Weigelt, 2017; Simcox & Fiez, 2014). As technology develops and new tools become available, the need to verify the compatibility between online and lab experiments increases.

Some labs have developed their own native mobile apps —downloadable from an app store (e.g., Google Play)— to run gamified versions of cognitive behavioral experiments (Brown et al., 2014; Dufau et al., 2011; Zimmerman et al., 2016). Others (e.g., Crump et al., 2013) have leveraged services such as Amazon Mechanical Turk (AMT) to reproduce classical experiments with AMT users, including four tasks that required detecting within-subject RT differences as short as 50 ms.

Recent studies have shown that online experiments are useful in numerical cognition research. An online study built in HTML/JavaScript (jsPsych (de Leeuw, 2015)) replicated classical RT effects in a task in which participants had to compare a single-digit number with a predetermined standard (Kochari, 2019). Huber et al. (2019) implemented a two-digit number comparison task on Qualtrics (Qualtrics, Provo, UT), and replicated previous findings, such as the decade distance effect. To avoid variability in RTs introduced by using different devices, data collected from mobile devices was discarded (a similar approach can be found in (Cipora et al., 2019) and (Gökaydin et al., 2018). However, as mobile devices are more widely used than computers, especially in emerging economies, this methodological approach may severely constrain the total number of potential participants in online experiments (Silver et al., 2019).

Despite deriving important lessons from past experiences using online tools for behavioral experiments, there are new challenges to take upon. First, in line with calls for open-science practices (Munafò et al., 2017), we ran an experiment with open-source tools and freely shared the code online. Second, we advocate for an integrated workflow encompassing experiment design and development as well as data analysis and modelling within a single developer’s framework (R in RStudio). While switching frameworks (and programming languages) according to the job at hand is certainly possible, it can be burdensome and error-prone (e.g., code bugs/errors due to syntax switching, different OS/software requirements). Furthermore, relevant tools from other environments (e.g., Python) can be used in R via an R version/wrapper (i.e., package). Third, to leverage the power of online experiments, we need a seamless and robust mobile user interface (i.e., an app that is intuitive, responsive, and reliable across OSs and web browsers), and adequate statistical models to account for device-related effects. Most published online studies fail to jointly meet all these requisites.

Native mobile apps are good to gamify experiments, but are usually task-specific and hard to adapt to new tasks. Moreover, programming these apps, as well as most web-based experiments, may require skilled coding in HTML, CSS, and JavaScript —languages not normally used by cognitive scientists for modeling and data analysis. While coding-free alternatives are available (e.g., jsPsych Builder [http://builder.jspsych.org], Gorilla [https://gorilla.sc/], PsychoPy [https://www.psychopy.org/], Lab.js [https://lab.js.org/], LabVanced [https://www.labvanced.com/]), some may not be free, open, and/or as flexible as the more “code-involved” options.

Shiny (Chang et al., 2018) is a package that runs on R, a programming language popular among cognitive researchers. It is well integrated in RStudio (a widely-used IDE to write and run R code). This package is relatively easy to learn and use for those familiar with scientific programming, and allows for experiment coding, data collection, data analysis and modelling, and creation of Markdown reports. Yet, despite its great potential to design and develop behavioral experiments as interactive web apps (Kaufman, 2020; Steiner et al., 2020), it remains underexploited in experimental psychology. Lastly, deploying a Shiny app online within RStudio is fairly simple: a newly developed app can be deployed online in minutes on the RStudio app-hosting service shinyapps.io. Quick app testing and sporadic data collection can be effectively done under a free plan. To circumvent limitations of the service’s free plan (CPU and memory limitations, participant cap), it is possible to install a Shiny-server on a cloud service or University server.

**Figure 1:**
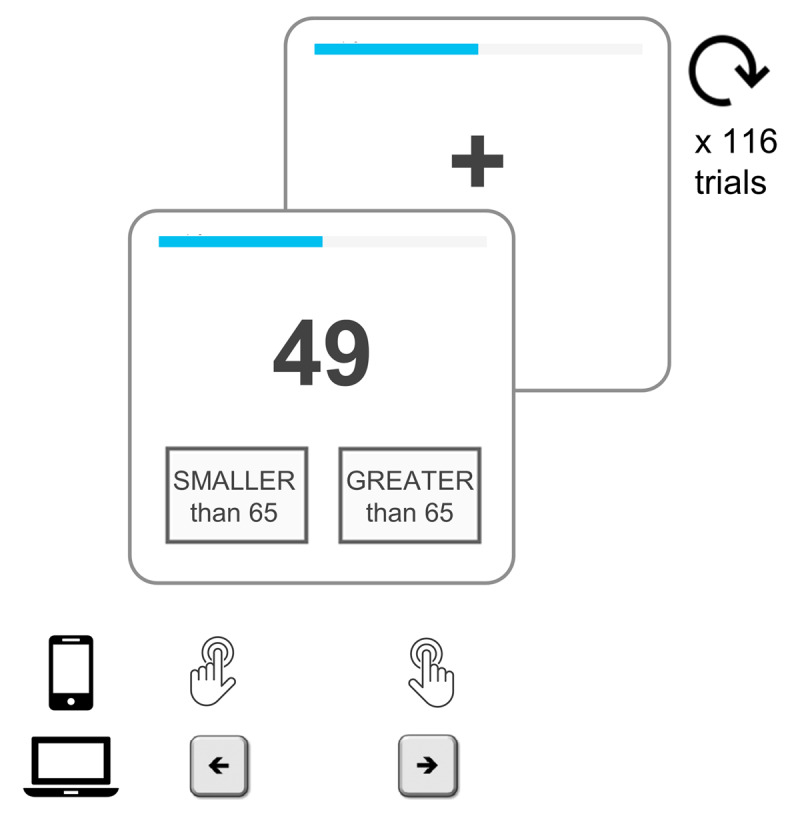
Number-comparison task. Participants had to decide whether a target number (49, in this case) was smaller or greater than a standard (65). We presented 58 target numbers: 29 were lower than the standard (from 36 to 64), and 29 were greater than the standard (from 66 to 94). Each of these targets was displayed twice, rendering a total of 116 trials. Trials were interleaved by an intertrial interval (ITI) that was randomly drawn from a uniform distribution (ITI ~ U(700 ms, 1000 ms)). Mobile device users responded on the screen by tapping a right or left box below the target, while desktop users responded by using the keyboard arrows. A left box tap/arrow keypress indicated a “smaller than 65” response and a right box tap/arrow keypress indicated a “greater than 65” response. On each trial, we recorded participants’ responses (smaller or greater) and response time (RT). Participants were instructed to be as accurate and fast as possible. To further enforce these instructions we applied a time deadline (each target was displayed for a maximum of 2 seconds) and a scoring system (+2 points for correct answers, –1 point for errors and timeouts). Finally, we included a progress bar (in light-blue at the top) to minimize dropouts due to uncertainty about how long the task would take.

Here, our main goal was to run a behavioral task as a Shiny app capable of measuring precise RTs at a millisecond range for participants using mobile devices, tablets and desktop computers. To this end, we implemented a two-digit number comparison task (***Figure 1***), in which participants had to decide whether a number (target) was smaller or larger than a reference number (standard). We relied on an already-developed R package (ShinyPsych (Steiner et al., 2020)) but customized some code to implement task-specific features. Typically, RTs are shorter and error rates are lower when the absolute difference between the numbers being compared is larger (i.e., comparing 61 vs. 65 is more difficult than comparing 34 vs. 65). This long-studied phenomenon, coined the numerical distance effect, has been used to shed light on how the mind represents numbers (Dehaene et al., 1990; Dehaene, 2011; Hinrichs et al., 1981; Krajcsi & Kojouharova, 2017; Moyer & Landauer, 1967; Nuerk et al., 2001; Verguts & De Moor, 2005).

This task was chosen due to three main reasons. First, it seems to reveal a universal feature of human numerical cognition (Dehaene, 2011), offering a robust benchmark to test our online tool. Indeed, several independent studies have found a linear mapping between RTs and logD —the logarithm of the absolute difference between the target (T) and the standard (S) numbers— with RTs decreasing about 100 ms per unit of logD (β_*logD*_ ≅ –100*ms*), even when the standard numbers were different (e.g. 55, 65, 75) (Dehaene, 1989; Dehaene et al., 1990; Hinrichs et al., 1981).

Second, since the numerical distance effect occurs in the millisecond range, its detection needs high-precision RT measurement —a usual requirement in experimental psychology studies (Plant, 2016; Ratcliff et al., 2010). Last, the task is arguably engaging and short, rendering it more suitable for online experimental settings.

Based on the above considerations we hypothesized that:

**H1:** RT decreases linearly with the logarithm of D, consistent with previous results (Dehaene, 1989, 2011; Dehaene et al., 1990; Hinrichs et al., 1981; Krajcsi & Kojouharova, 2017; Moyer & Landauer, 1967, 1973; Nuerk et al., 2001; Verguts & De Moor, 2005).

**Figure 2:**
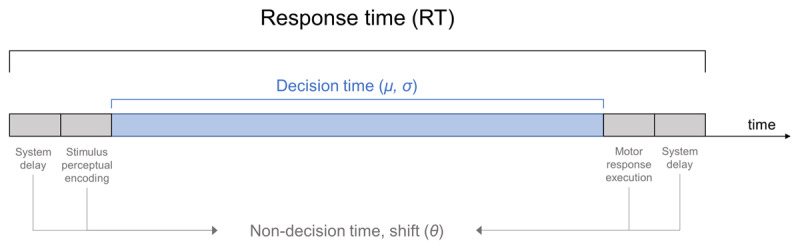
Temporal decomposition of the recorded response time (RT). On each trial, the recorded RT is the sum of a decision time and a non-decision time or shift (*θ*). The shift is assumed to represent both internal processes (perceptual encoding of the stimulus and motor response execution) and external delays. The latter are exclusively technological and include delays due to hardware, OS and browser processing speed, such as the latency between button, keypad or touchscreen press to the actual recording of the response. The decision time is the time the participant takes to evaluate the evidence, and it is assumed to follow a log-Normal distribution with parameters *μ* and *σ*.

To maximize recruitment, online experiments are often designed for multiple devices, meaning RT data will have several sources (e.g., iPad, Android phone). Importantly, measured RTs not only represent the time needed to make a decision, but they also include shifts (i.e. delays in recorded RTs) due to either internal processes (perceptual encoding of the stimulus and motor response execution) or external sources (***Figure 2***). Specifically, each type of device might introduce a different delay related to hardware, OS and/or browser processing speed (Anwyl-Irvine et al., 2020; Bridges et al., 2020). Indeed, mobile devices have larger shifts than desktop computers (Holden et al., 2019; Pronk et al., 2020; Reimers & Stewart, 2008). To control for device-specific variability, we collected participants’ device information (OS and web browser), incorporated this information into a combined variable in a statistical model, and determined its impact on RT. We therefore hypothesized that:

**H2:** Participants’ device, OS, and web browser introduce systematic delays in recorded RTs. Specifically, we expected that mobile devices would introduce larger shifts on RTs than desktop devices.

## Method

### Participants

Participants accessed the app online via a shortened URL link distributed across Facebook, Twitter, and WhatsApp. This link redirected them to the Shiny server hosting the app (Donweb [donweb.com] cloud server with 2GB of memory). Our aim was collecting data from at least 84 participants, twice the sample in Dehaene et al., 1990 (n = 42), as our task had half the number of trials. Within the first week of having the app online, we surpassed this minimum sample size and stopped data collection. A total of 169 participants (65 males, age range: 18–72 y.o., mean age: 31.94 ± 11.69 y.o.) completed the online task either with a mobile device (n = 109) or on a desktop computer (n = 60). All participants gave informed consent, and the local ethics committee approved the study (“Centro de Educación Médica e Investigaciones Clínicas” protocol ID 435). No monetary compensation was awarded to participants.

### Task

In this two-digit number comparison task, adapted from Dehaene et al., 1990, participants had to decide whether a target number was smaller or greater than 65. We presented 58 target numbers: 29 were lower than the standard (from 36 to 64), and 29 were greater than the standard (from 66 to 94) (***Figure 1***). Each target was displayed twice, yielding a total of 116 trials. These were pseudo-randomized so that the same target was never displayed in two consecutive trials and the correct response (greater or lower than the standard) was never the same for more than two consecutive trials. We constructed 19 different lists of numbers with these features, and one was randomly selected at the beginning of each task-session. An intertrial interval (ITI) randomly drawn from a uniform distribution, ITI ~ U(700 ms, 1000 ms) was imposed.

In each trial, a target number was presented in bold Arial font (font size 113 for mobile devices, 165 for desktop) for a maximum of 2 seconds. Desktop users responded by using the keyboard arrows, while mobile device users responded on the screen by tapping a right or left box below the target. The opacity of these boxes changed on-response as visual feedback that a response had been recorded. A left box tap/arrow keypress indicated a “<65” response, and a right box tap/arrow keypress indicated a “>65” response. We recorded RTs and response type (smaller or greater) for each trial.

### Programming in Shiny

Task implementation was largely based on ShinyPsych (Steiner et al., 2020), a recently-developed R package for designing behavioral experiments on Shiny (Chang et al., 2018). This package was selected because each element in the app flow (instructions, training, experimental phase, demographic survey, and debriefing) is built as a separate “page” that is rendered when users interact with the app. Furthermore, ShinyPsych offers simple functions to specify elements within a page and includes ready-to-use templates (which we leveraged for the demographic survey). However, since ShinyPsych did not support specific aspects of our task (e.g., flexible implementation across devices and systems), we further customized the app via R/HTML/CSS/JavaScript. We included the following features: collection of device type (mobile or desktop computer), web browser and OS; variable ITIs in which a fixation cross was displayed; scoring system; task progress bar; device-dependent visual parameters (e.g., text size); device-dependent responses (key-pressing for desktop and box-tapping for mobile users); and RT collection (using differences in browser timestamps via the *Date.now()* JavaScript function) with a temporal deadline (upon which a custom timed-out message popped-out and remained on screen for a fixed amount of time). Importantly, the latter offers reliable time measurements, which is critical to infer the numerical-distance effect from RT data. Our app (as any Shiny app) uses JavaScript under the hood to record events and user-interactions within an HTML page. Specifically, RT is defined as the interval between two specific events: stimulus (number) onset and user response. RTs were obtained through JavaScript functions that counted the time elapsed from number onset to user-response (box-tap or key-press, for mobile and desktop devices, respectively).

### Procedure

Participants clicked on a link distributed across social media platforms to access the online app. Upon connection, instructions were displayed, and informed consent was prompted. As in Dehaene et al., 1990, we instructed participants to be as accurate and fast as possible. To further enforce these instructions, we applied a time deadline (2 seconds) and a scoring system (+2 points for correct answers, –1 point for errors and timeouts). This scoring system also served as a way to “gamify” the app and promote engagement in the task (Lieberoth, 2015; Lumsden et al., 2016; Wiley et al., 2020). Users could track their progress through a progress bar, which also aided in reducing dropouts for not knowing when the task would end.

The experimental phase started after four training trials. After the task, participants reported their age, sex, and education level on an in-app survey built with the ShinyPsych package (the survey can be found at the project’s OSF repository: https://osf.io/9w28t/). Finally, performance feedback (total points) was provided and a short debriefing text was shown.

Sessions lasted 7 minutes on average, with the shortest lasting 4 minutes.

### Data Analysis & Statistical Models

To determine whether our results support the numerical distance effect, rather than relying on ANOVA and OLS regressions as in Dehaene et al., 1990, we based our analyses on generalized linear mixed models (GLMM) that were estimated with Bayesian methods. We applied GLMMs because they extend the general linear models to the exponential family of probability distributions, which include the shifted log-Normal and Bernoulli probability density functions that we used to model RT and error rate, respectively. Moreover, random-effects (multi-level) modeling allowed us to represent within-subject data-dependency through a grouping variable which, at the same time, increased model parsimony and allowed for generalization in inference and interpretation. We created our GLMMs within the Stan computational framework (Carpenter et al., 2017) accessed with *brms* (Bürkner, 2017), which uses a No-U-Turn Sampler (NUTS, an extension of the Hamiltonian Monte Carlo sampling method) to estimate posterior probabilities. To improve convergence and prevent overfitting, we used weakly informative —yet regularizing— priors (Gelman et al., 2008).

Our modelling strategy followed a sequential rationale stemming from *Bayesian workflow* guidelines (Gelman et al., 2020): hypothesis-driven model specification and descriptive data-analysis, prior predictive checking (to assess our priors’ suitability), estimation, diagnosis (using the *launch_shinystan* function from the *shinystan* package (Gabry, 2018) to inspect —among other diagnostics— sampling convergence and autocorrelation), validation (via posterior predictive checks), and inference (i.e., hypothesis testing).

Next, we report the model specification for the RT and error rate models.

#### Response times

As shown in previous studies on numerical distance, the main predictor of mean RT is the log of the absolute difference between the target (T) and the standard (S) numbers, *D = abs*(*T-S*), i.e. RT ~ logD (Dehaene et al., 1990; Hinrichs et al., 1981; Nuerk et al., 2001). However, as RTs are positive, right-skewed, and necessarily greater than the time required to encode stimuli, RTs were modeled as a shifted-logNormal random variable (Wagenmakers & Brown, 2007):


\log \left({RT-\theta} \right)\sim Normal\left({\mu,\sigma} \right)

This probability density function is defined by three parameters: *μ* (mean), *σ* (SD) and *θ* (non-decision time or shift). The latter accounts for both psychological (stimulus perceptual-encoding and motor response execution), and technological components (CPU, OS, and web browser) (***Figure 2***). To render a linear relationship between mean RT and logD, we model *μ* as the logarithm of a linear predictor (*ν*), which includes, among others —as we will detail below—, the coefficient for logD.


RT\sim{e^{Normal\left({\log \left(\nu \right),\sigma} \right)}}+\theta

This way, mean RT maps linearly onto logD, i.e., E[RT] = *ν*, and the coefficients from the linear predictor of *ν* can be interpreted analogously to those of previous studies.

Beyond our focus on the numerical distance effect, we also examined whether the mean, standard deviation and/or shift of RT were affected by device type, OS and web browser. To this end, the model included a predictor variable that collectively represents these features of the participant’s interface that we called *system*. Altogether, the predictor variables for *ν, σ*, and *θ* were:

*logD*: A numerical variable indicating the log of the absolute numerical distance between the target presented on each trial and the standard (values of logD range from log(1) = 0 and log(29) ≅ 3.37).

*smaller*: A categorical variable indicating whether the target in a given trial was smaller (1) or greater (0) than the standard — this is relevant because previous studies show that numerical comparisons are slower for smaller than for greater numbers (Dehaene et al., 1990).

*system*: A categorical variable indicating the participant device’s OS and web browser. Our data contained 10 categories: four from mobile devices (Android.Chrome, iPhone.Mozilla, iPhone.Safari and iPad.Mozilla) and six from desktop computers (Windows.Chrome, Windows.Firefox, Linux.Chrome, Linux.Firefox, Macintosh.Chrome and Macintosh.Safari). These categories are indexed with sub-index *j* (j = 1,…,9), and “Android.Chrome” was used as the reference category.

*trial.number*: A numerical variable, ranging from 1 to 116, that indicates trial number (centered and scaled) within each participant’s session, to control for chronological effects (e.g., fatigue, learning, hurry-to-finish).

*age*: A numerical variable (centered and scaled), ranging from 18 to 72, to control for participants’ age effects.

Next, we report the specification for the linear predictors of *ν, σ*, and *θ*. To aid model convergence, we used a log-link function for the linear predictors of *σ* and *θ*.


1 \begin{array}{l}
{\nu _i} = {\beta _{Intercept,{{\rm id}_i}}} + \\
\,\,\,\,\,\,\,\,\,\,{\beta_{trial.number, {\rm id}_i}}\times {\bf trial.number}_{\boldsymbol i} + \\
\,\,\,\,\,\,\,\,\,\,{\beta_{smaller, {\rm id}_i}} \times {\bf smaller}_{\boldsymbol i} + \\
\,\,\,\,\,\,\,\,\,\,{\beta_{{\rm log}D, {\rm id}_i}} \times {\bf logD}_{\boldsymbol i} + \\
\,\,\,\,\,\,\,\,\,\,{\beta_{age}} \times {\bf age}_{\boldsymbol i} + \\
\,\,\,\,\,\,\,\,\,\,{\beta_{system, j}} \times {\bf system}_{{\boldsymbol i}, {\boldsymbol j}} + \\
\,\,\,\,\,\,\,\,\,\,{\beta_{{\rm log}D}} \times {\bf logD}_{i} \times {\bf smaller}_{\bf i}
\end{array}

2 \begin{array}{l}
\log \left({{\sigma _i}} \right) = \,{\gamma _{Intercept}}\, + \\
\,\,\,\,\,\,\,\,\,\,\,\,\,\,\,\,\,\,\,\,\,\,\,\,\,{\gamma _{system,\,j}}\, \times \,\,{\bf system}_{{\boldsymbol i}, {\boldsymbol j}}\, + \\
\,\,\,\,\,\,\,\,\,\,\,\,\,\,\,\,\,\,\,\,\,\,\,\,\,\,{\gamma _{age}}\, \times {\bf age}_{\boldsymbol i}\,
\end{array}

3 \begin{array}{l}
\log \left({{\theta_i}} \right) = {\delta _{Intercept, id_{i}}}\, + \\
\,\,\,\,\,\,\,\,\,\,\,\,\,\,\,\,\,\,\,\,\,\,\,\,\,{\delta_{system, j}} \times {\bf system}_{{\boldsymbol i}, {\boldsymbol j}} + \\
\,\,\,\,\,\,\,\,\,\,\,\,\,\,\,\,\,\,\,\,\,\,\,\,\,\,{\delta_{age}} \times {\bf age}_{\boldsymbol i}
\end{array}

Note: *id*: participant ID; *i*: trial index; *j*: system index.

We represented the hierarchical structure of the data (i.e., the within-subjects design) by modelling random effects by participant ID (*id*) for the intercept of the linear predictor for *ν* and *θ*, and over the coefficients for the effects of *logD, smaller*, and *trial.number* on *ν*. Moreover, we modeled the correlation between the random effects and these coefficients, and between the intercepts of *ν* and *θ*. The corresponding covariance and correlation matrices are as follows.


\begin{array}{l}
\left[{\begin{array}{*{20}{c}}
{{\beta _{Intercept,\,id}}}\\
{{\delta _{Intercept,\,id}}}
\end{array}} \right]\sim MVNormal\left({\left[{\begin{array}{*{20}{c}}
{{\beta _{Intercept}}}\\
{{\delta _{Intercept}}}
\end{array}} \right],\,{S_{Intercept}}} \right)\\
{S_{Intercept\,}} = \,\left({\begin{array}{*{20}{c}}
{{\sigma _{{\beta _{Intercept}}}}}\\ 0
\end{array}\begin{array}{*{20}{c}} 0\\
{{\sigma _{{\delta _{Intercept}}}}}
\end{array}} \right)\,\, \times \,\,{R_{Intercept}}\, \times \,\,\left({\begin{array}{*{20}{c}}
{{\sigma _{{\beta_{Intercept}}}}}\\ 0
\end{array}
\begin{array}{*{20}{c}} 0\\
{{\sigma _{{\delta _{Intercept}}}}}
\end{array}} \right)\\
\left[\begin{array}{l}
{\beta _{trial.number,\,id}}\\
\,\,\,\,{\beta _{smaller,\,id}}\\
\,\,\,\,\,\,\,{\beta _{{\rm log}D,\,id}}
\end{array} \right]\,\,\sim\,\,MVNormal\,\,\left({\left[\begin{array}{l}
{\beta _{trial.number}}\\
\,\,\,\,{\beta _{smaller}}\\
\,\,\,\,\,\,\,{\beta _{{\rm log}D}}
\end{array} \right],\,{S_\beta }} \right)\\
{S_\beta }\, = \,\left({\begin{array}{*{20}{c}}
{{\sigma _{{\beta _{trial.number}}}}}& 0& 0\\
0& {{\sigma _{{\beta _{smaller}}}}}& 0\\
0& 0& {{\sigma _{{\beta _{{\rm log}D}}}}}
\end{array}} \right)\,\, \times \,\,{R_\beta }\, \times \,\,\left({\begin{array}{*{20}{c}}
{{\sigma _{{\beta _{trial.number}}}}}& 0& 0\\
0& {{\sigma _{{\beta _{smaller}}}}}& 0\\
0& 0& {{\sigma _{{\beta _{{\rm log}D}}}}}
\end{array}} \right)
\end{array}

Note: *MVNormal*: Multivariate Normal; *S*: covariance matrix; *R*: correlation matrix.

Next, we report prior specifications for all the estimated model parameters. These are weakly-informative priors that generate plausible data (assessed with prior predictive checks).


\begin{array}{l}
{\beta _{Intercept}}\,\sim \,Normal\left({0.6,\,0.3} \right)\\
\left({{\beta _{trial.number}},\,{\beta _{smaller}},\,{\beta _{{\rm log}D}},\,{\beta _{system, j}},\,{\beta _{{\rm log}D\, \times \,smaller}}} \right)\,\sim\,Normal\left({0,\,1} \right)\\
\\
{\gamma _{Intercept}}\,\sim\,Normal\left({-1,\,\,0.2} \right)\\
\left({{\gamma _{system, j}},\,{\gamma _{age}}} \right)\,\sim\,Normal\left({0,\,\,0.1} \right)\\
\\
{\delta_{Intercept}}\,\sim\,Normal\left({-1.3,\,\,0.2} \right)\\
\left({{\delta _{system, j}},\,{\delta _{age}}} \right)\,\sim\,Normal\left({0,\,\,0.1} \right)\\
\\
\left({{\sigma _{{\beta _{Intercept}}}},\,{\sigma _{{\beta _{trial.number}}}},\,{\sigma _{{\beta _{smaller}}}},\,{\sigma _{{\beta _{{\rm log}D}}}}} \right)\,\sim\,Normal\left({0.2,\,0.2} \right)\\
{\sigma _{{\delta _{Intercept}}}}\,\sim\,Student\left({3,\,\,0,\,\,2.5} \right)\\
\\
\left({{R_{Intercept}},\,{R_\beta }} \right)\,\sim\,LKJcorr\left(1 \right)
\end{array}

#### Error rate

We modeled errors as a Bernoulli random variable (i.e., a binary categorical variable where 1 = Error and 0 = Correct) with a logit link to the linear predictor of the probability *π* of making an error (Equation (4) below), which included as predictor variables *trial.number* and *logD*. In other words, for each trial *i*, we estimated the probability *π*_i_ of making an error on that trial, conditional on these predictor variables.


4 \begin{array}{l}
Erro{r_i}\sim\,Bernoulli\left({{\pi _i}} \right)\\
{\rm logit}\left({{\pi _i}} \right) = \,{\varphi _{Intercept,\,i{d_i}}}\, + \\
\,\,\,\,\,\,\,\,\,\,\,\,\,\,\,\,\,\,\,\,\,\,\,\,\,\,\,\,{\varphi_{trial.number,\,i{d_i}}}\, \times \,{\bf trial.number}_{\boldsymbol i}\, + \\
\,\,\,\,\,\,\,\,\,\,\,\,\,\,\,\,\,\,\,\,\,\,\,\,\,\,\,\,{\varphi_{smaller,\,i{d_i}}} \times \,{\bf smaller}_{\boldsymbol i}\, + \\
\,\,\,\,\,\,\,\,\,\,\,\,\,\,\,\,\,\,\,\,\,\,\,\,\,\,\,\,{\varphi_{{\rm log}D,\,i{d_i}}} \times \,{\bf logD}_{\boldsymbol i}\, + \\
\,\,\,\,\,\,\,\,\,\,\,\,\,\,\,\,\,\,\,\,\,\,\,\,\,\,\,\,{\varphi_{age}} \times \,{\bf age}_{\boldsymbol i}\, + \\
\,\,\,\,\,\,\,\,\,\,\,\,\,\,\,\,\,\,\,\,\,\,\,\,\,\,\,\,{\varphi_{system,\,j}} \times \,{\bf system}_{{\boldsymbol i}, {\boldsymbol j}}\, + \\
\,\,\,\,\,\,\,\,\,\,\,\,\,\,\,\,\,\,\,\,\,\,\,\,\,\,\,\,{\varphi_{{\rm log}D \times \,smaller}} \times \,{\bf logD}_{\boldsymbol i} + {\bf smaller}_{\boldsymbol i}
\end{array}

Similarly to the RT model, we represented the hierarchical structure of the data by modelling random effects by participant ID (*id*) for the intercept and the *trial.number* coefficient of the linear predictor for *π*, and modeled the correlation between these random effects. Next, we show how we represented these relations.


\begin{array}{l}
\left[ \begin{array}{l}
\,\,\,{\varphi _{Intercept,\,id}}\\
{\varphi _{trial.number,\,id}}\\
\,\,\,{\varphi _{smaller,\,id}}\\
\,\,\,\,\,\,{\varphi _{{\rm log}D,\,id}}
\end{array} \right]\,\,\sim\,\,MVNormal\left({\,\left[\begin{array}{l}
\,\,{\varphi_{Intercept}}\\
{\varphi_{trial.number}}\\
\,\,\,{\varphi_{smaller}}\\
\,\,\,\,\,\,{\varphi_{{\rm log}D}}
\end{array} \right], S} \right)\\
S = \left[{\begin{array}{*{20}{l}}
{{\sigma_{{\varphi_{Intercept}}}}} & 0 & 0 & 0\\
0 & {{\sigma_{{\varphi_{trial.number}}}}} & 0 & 0\\
0 & 0 & {{\sigma_{{\varphi_{smaller}}}}} & 0 & \\
0 & 0 & 0 & {{\sigma _{{\varphi_{{\rm log}D}}}}}
\end{array}} \right] \times R \times \left[{\begin{array}{*{20}{l}}
{{\sigma_{{\varphi_{Intercept}}}}} & 0 & 0 & 0\\
0 & {{\sigma_{{\varphi_{trial.number}}}}} & 0 & 0\\
0 & 0 & {{\sigma_{{\varphi_{smaller}}}}} & 0\\
0 & 0 & 0 & {{\sigma_{{\varphi_{{\rm log}D}}}}}
\end{array}} \right]
\end{array}

Next, we report prior specifications for all the estimated model parameters.


\begin{array}{l}
\left({{\varphi _{Intercept}}, {\varphi _{trial.number}}, {\varphi _{smaller}}, {\varphi _{{\rm log}D}}, {\varphi _{age}}, {\varphi _{system, j}}, {\varphi_{{\rm log}D \times smaller}}} \right) \sim Normal\left({0, 1} \right)\\
\left({{\varphi _{Intercept}}, {\varphi _{trial.number}}, {\varphi _{smaller}}, {\varphi _{{\rm log}D}}} \right) \sim Student \left({3, 0, 2.5} \right)\\
R \sim LKJcorr\left(1 \right)
\end{array}

To diagnose model estimation, we (i) evaluated trace autocorrelation and number of effective samples, (ii) visually inspected chains with trace plots for each model parameter, and (iii) assessed whether \hat R values were smaller than 1.01 (denoting that chains mixed well) using *launch_shinystan* (from the *shinystan* package (Gabry, 2018)). For both models, we drew 20,000 samples (4 chains of 6,000 traces each with the first 1,000 for warm up) with an adaptive delta of 0.99 to minimize divergent transitions after warm up. As a result, there were zero divergent transitions during sampling of both models.

**Figure 3:**
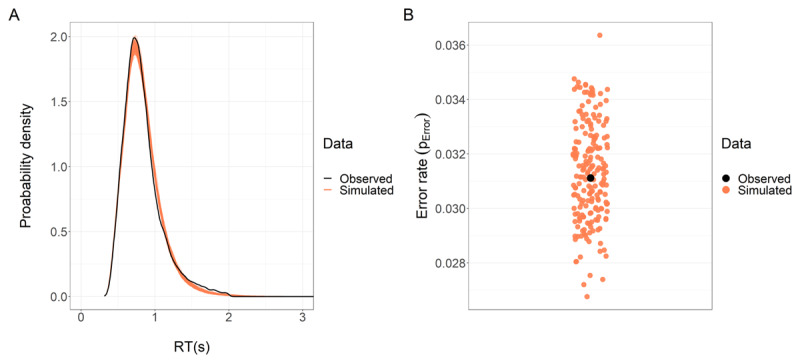
Posterior predictive checks for the RT and error rate models. **(A)** We simulated 200 datasets with the same structure as the observed dataset, using the RT model posterior distribution. We plotted the distribution of RT for each of these 200 simulations (light-red lines) and the observed RT distribution (black line). The high degree of overlap between the simulated and observed data distributions is indicative of the model’s ability to capture the distributional features of the observed RTs. **(B)** We performed a similar analysis for the error rate model but, since this variable is not continuous, we computed the mean error rate for each of the 200 simulated datasets (light-red dots) and for the observed data (black dot). Mean error rates from the simulated datasets are scattered around the observed error rate mean, which is indicative of the model’s ability to capture the main distributional feature of the observed error rate.

We validated the models with posterior predictive checks (PPCs) by simulating 200 new datasets from the estimated parameters’ posterior distributions, and overlaying the simulated distributions over the observed data distribution. PPCs allowed us to understand whether (and to what degree) the estimated model captured distributional signatures of the observed data. For both models, the posterior predictive distributions were qualitatively indistinguishable from the observed distributions (***Figure 3***). To quantify this qualitative assessment, we computed —for the error model— the proportion of errors and -for the RT model- the 0.1, 0.3, 0.5 (median), 0.7, and 0.9 quantiles and SD of the conditional (by *smaller*, and by *system*) RT distributions of each set. We then obtained the median and the quantile-based credible intervals (containing 95% of the probability density, CI_95_) of the distribution of these summary-values and compared them to the observed-data summaries (assessing whether the observed value was contained in the CI_95_). This approach assigned credibility to the model while offering a measure of uncertainty from the distribution of simulated summary statistics. For the error rate model, the CI_95_ of the conditional summary statistics included all the summary statistics of the observed data. Similarly, for the RT model, this was generally true, except that -mainly- for the 0.9 quantile for some categories of system, the simulations were off by a maximum of 10 ms from the observed summary statistic.

Additionally, we computed a measure of in-sample goodness-of-fit for each model. For the RT model, we computed a Bayesian approximation to R^2^ (Gelman et al., 2019) and found that R^2^ = 0.438 (CI_95_ = [0.429, 0.447]). For the error rate model, we computed the area under the Receiver Operating Characteristic curve (AUC-ROC), which reflects the model’s classification ability (as a rule-of-thumb, AUC-ROC = 0.8 denotes a good model performance (Hosmer Jr et al., 2013)), and found that AUC-ROC = 0.813.

Finally, we tested our hypotheses directly over the posterior probability distribution by computing the CI_95_ of the coefficient-of-interest distribution and assessing whether this interval contained (or for the logD predictor, was smaller than) 0. If the CI_95_ did not include 0 (or if 95% of the density was smaller than 0, for logD), the results support the hypothesis that the corresponding coefficient is different from (smaller than) that null value. To aid interpretation of the error rate model, we first exponentiated the coefficient-of-interest posterior values to obtain odds ratios, and then computed the summary values of these posterior values (median and CI_95_).

## Results

**Figure 4:**
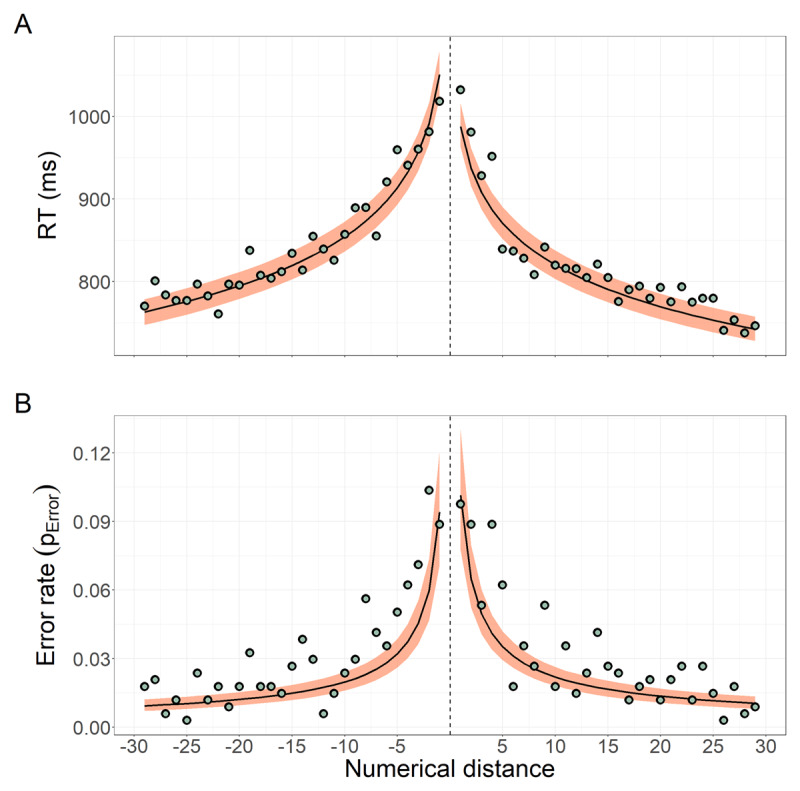
Numerical distance effect on RT and error rate. (**A**) Expected and observed values for RT as a function of the numerical distance between the target and the standard (distance is 0 at the dashed vertical line). Green dots are the observed overall mean of RT for each distance. The black line and shaded area represent the median and CI_95_ of the RT expected values (for an average participant), respectively, obtained from 2,000 model posterior samples. (**B**) Expected and observed values of error rate (i.e., probability of making an error) as a function of the numerical distance. Green dots are the overall proportion of errors for each distance. The black line and shaded area represent the median and CI_95_ of the expected values (for an average participant), respectively, obtained from 2,000 model posterior samples. Note that, as not all systems were equally represented in our data (e.g., there was only one session from an iPad device), we obtained and represented the weighted (by the frequency of the participants’ systems) marginal means for each distance in both panels. All means were computed for mean participants’ age and at mean trial number (i.e., at the middle of the task).

We found evidence consistent with a numerical-distance effect on RT. As expected, the greater the absolute difference between target and standard, the faster the responses (***Figure 4A***). Regression coefficients for our model (Equations 1–3) show that, on average, RTs decrease 70 ms for every unit increase in logarithmic numerical distance, e.g. roughly the distance from 66 (log_(D = 1)_ = 0) to 68 (log_(D = 3)_ ≅ 1) (β_*logD*_ = –70.3*ms, CI*_95_ = [–75.4, –65.6]*ms*). Strikingly, our estimated value of β_*logD*_ closely resembles that estimated from the data shown in Dehaene et al., 1990^1^ (β_*logD* (*Dehaene et al.,1990*)_ = –78.3*ms*), even when we used a different statistical model for RT and different technology to implement the task. Also consistent with previous studies (Dehaene, 1989; Dehaene et al., 1990; Hinrichs et al., 1981), we found that RTs for numbers smaller than 65 were slower than RTs for numbers greater than 65 (β_*smaller*_ = 55.8*ms, CI*_95_ = [39.7,72.3]*ms*). Again, our estimated value for β_*smaller*_ closely matches that from Dehaene et al., 1990 (β_*smaller* (*Dehaene et al.,1990*)_ = 53.8*ms*).

We detected chronological effects on RT by which for every 35 completed trials, correct responses were made on average 20 ms faster (β_*trial.number*_ = –20.6*ms, CI*_95_ = [–26.1, –15.3]*ms*). Further, we found that older participants were predominantly slower than younger ones (β_*age*_ = 10.1*ms, CI*_95_ = [–2.18, 22.4]*ms*). This value corresponds to an average increment of ~9 ms for every 10 years of age. Indeed, the impact of age over RTs is a well-documented effect in binary RT decision-making tasks (Ratcliff et al., 2004).

**Figure 5:**
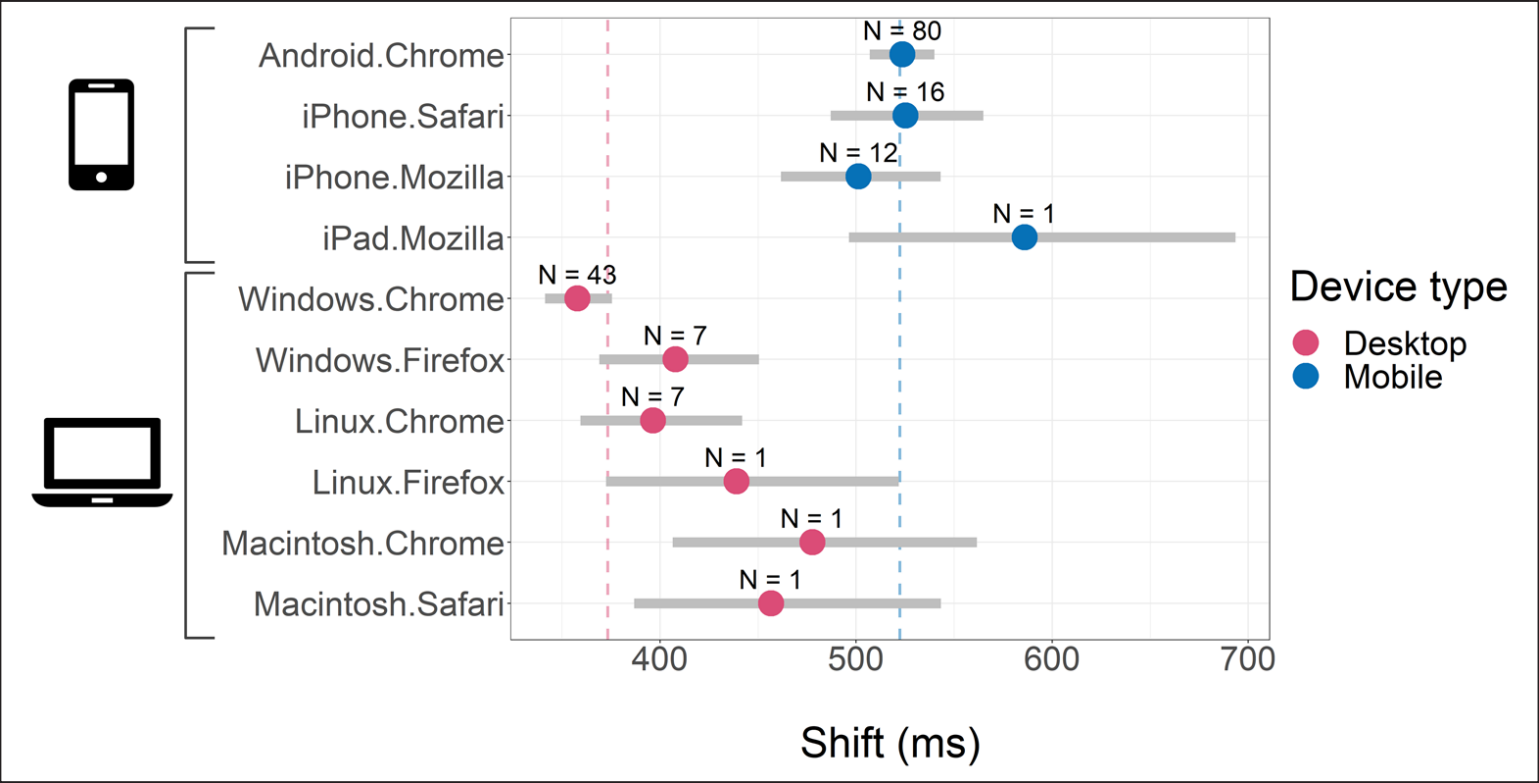
Shifts by participants’ system. We computed the conditional shift (*θ*) means by system (i.e., for each of the 10 categories of systems, see Methods) using 2,000 posterior samples from the estimated coefficients of the linear predictor of *θ* (Equation (3)). The colored points’ position and grey horizontal intervals represent the median and CI_95_ of the posterior samples for each conditional mean, respectively. We used dark pink and blue points to represent mobile and desktop devices, respectively, to show that mobile devices induce larger shifts than desktop devices. The annotation above each point reports the number of participants (N) that completed the task using that specific system. The dark pink and blue vertical dashed lines in the background are the shift means for mobile and desktop devices (weighted by the frequency of the participants’ systems, for each device type), respectively. All means were computed for mean participants’ age.

Our modeling strategy also allowed us to understand whether and how the participants’ system (i.e. device, OS, and web browser) affected their RTs. We found that the system indeed affected shift (*θ*) (***Figure 5***) but we did not find evidence for an effect of the system on *ν* nor on *σ* of RTs (***Table 1***). This means that variability associated with different user device types affected the non-decision portion of the measured RTs (modeled as a component of *θ*) but did not affect the mean or standard deviation of the decision portion of the RTs (modeled as *ν* and *σ* of the decision portion of the RTs, respectively). The main pattern we observed is that RTs from mobile devices (mainly Chrome browser running on Android OS) had larger shifts (*θ*) than RTs from desktop computers (mainly Chrome browser running on Windows OS) (Δθ_*mobile-desktop*_ = 149*ms, CI*_95_ = [128, 168]*ms*), which is consistent with previous reports (Holden et al., 2019; Pronk et al., 2020; Reimers & Stewart, 2008).

**Table 1:** *Estimated Effects of the Participants’ System Over ν and σ*.


Distributional parameter	Coefficient	Median	Lower	Upper

*ν*	*β*_system, Windows.Chrome_	–0.009	–0.037	0.019

*ν*	*β*_system, Windows.Firefox_	–0.030	–0.086	0.025

*ν*	*β*_system, Linux.Chrome_	–0.041	–0.097	0.015

*ν*	*β*_system, Linux.Firefox_	0.006	–0.130	0.141

*ν*	*β*_system, Macintosh.Chrome_	0.010	–0.126	0.148

*ν*	*β*_system, Macintosh.Safari_	0.025	–0.125	0.178

*ν*	*β*_system, iPhone.Safari_	–0.001	–0.043	0.042

*ν*	*β*_system, iPhone.Mozilla_	–0.012	–0.057	0.033

*ν*	*β*_system, iPad.Mozilla_	–0.061	–0.210	0.095

*σ*	*γ*_system, Windows.Chrome_	–0.010	–0.059	0.039

*σ*	*γ*_system, Windows.Firefox_	0.037	–0.052	0.123

*σ*	*γ*_system, Linux.Chrome_	–0.054	–0.140	0.031

*σ*	*γ*_system, Linux.Firefox_	–0.090	–0.229	0.047

*σ*	*γ*_system, Macintosh.Chrome_	–0.049	–0.202	0.100

*σ*	*γ*_system, Macintosh.Safari_	0.092	–0.051	0.234

*σ*	*γ*_system, iPhone.Safari_	0.044	–0.027	0.111

*σ*	*γ*_system, iPhone.Mozilla_	–0.007	–0.088	0.069

*σ*	*γ*_system, iPad.Mozilla_	0.119	–0.059	0.283

*Note*: Lower and Upper refer to the lower and upper bounds of the CI_95_ of each coefficient posterior distribution. Importantly, all these intervals contain 0, suggesting that the participants’ system did not have a relevant effect on either *μ* or *σ* of RTs.

Error rates were also revealing of a numerical distance effect, by which the greater the numerical distance, the smaller the rate of errors. In line with previous studies (Dehaene et al., 1990; Hinrichs et al., 1981), we observed that participants made ~10% errors for the hardest numerical comparisons (e.g., 64 vs 65, 66 vs 65), and that the error rates dropped as the numerical distance increased (***Figure 4B***). Specifically, we found that for each unit increase in logarithmic numerical distance, the odds of making an error decreased by 50% on average (φ_*logD*_ = –0.71, *CI*_95_ = [–0.80, –0.62]).

Although the task was rather short (116 trials), we found a relevant chronological effect (φ_*trial.number*_ = –0.15, *CI*_95_ = [–0.27, –0.03]). This implies that for —roughly— every 35 completed trials, the odds of making an error decreased by 15%. This effect possibly represents a learning process by which fewer errors are made as the task unfolds.

To summarize, data collected in an online experiment is adequate to detect the numerical-distance effect on RT and error rate. Notably, the magnitude of this effect closely matches previous results. Moreover, we found that the system that participants used to complete the task (i.e., the OS and web browser of their devices) reliably affected shifts, but we did not find evidence for changes in the mean nor the SD of RTs. We quantified the change in shift for each different system and found that mobile devices generally induced larger shifts than desktop systems.

## Discussion

Many paradigms in cognitive science rely on measuring RTs at the millisecond-range. Collecting behavioral data online is attractive but methodologically challenging. Since participants will use different OSs, web browsers, and devices, in potentially distracting contexts, online experiments may resemble the setting of an uncontrolled environment.

In this paper, we show that high quality data (including precise RTs) in binary decisions can be obtained with an R-Shiny application running on mobile devices and desktop systems. As a proof-of-principle, we replicated the well-established numerical distance effect, typified by decreased RTs and error rates when the absolute difference between compared numbers increases (Dehaene et al., 1990; Hinrichs et al., 1981; Krajcsi & Kojouharova, 2017; Moyer & Landauer, 1967; Verguts & De Moor, 2005).

Behavioral studies reported 35–40 years ago found that RTs map linearly onto the log of the numerical distance (logD) with a regression coefficient of β_*logD* (*Dehaene et al., 1990*)_ = –78.3*ms* when the comparison task uses 65 as the standard (Dehaene et al., 1990) with small variations when the standard is set to 55 or 75 (Dehaene, 1989; Dehaene et al., 1990; Hinrichs et al., 1981). Considering the huge technological and statistical changes occurring in the last four decades, it was far from obvious that such findings could be replicated in an online experiment involving fairly uncontrolled conditions.

The precise cognitive mechanisms underlying this phenomenon remain a matter of debate (Dehaene, 1989; Dehaene et al., 1990; Hinrichs et al., 1981; Krajcsi & Kojouharova, 2017; Moyer & Landauer, 1973; Nuerk et al., 2001; Verguts & De Moor, 2005). While our work was not aimed to discern between these accounts, it contributes to the literature by showing that reliable RTs can be measured using R-Shiny, amplifying data collection options for the field.

Our results also allowed us to identify differences in shift (*θ*) due to participants’ hardware and software such as web browser and OS. We collected data from 10 different combinations of OS, web browser, and type of device (mobile or desktop) that we collectively called *system*. However, these categories were not equally represented in our data. In fact, most participants completed the task using Chrome browser (running either on Android [mobile] or Windows [desktop] OS), limiting the interpretation of our results to comparisons between such systems. Nonetheless, we found that mobile devices have shifts that are ~150ms larger than those of desktop computers. These differences might reflect OS and browser processing times, the response mode (box-tap for mobile devices, keypress for desktops), and/or hardware limitations. Moreover, by modelling system-specific contributions to shifts we were able to control for device-dependent RT variability. While such variability may not constitute a confound in within-subjects designs, it is critical to address it in between-subjects designs.

In the last decade, many tools have been developed to perform experiments online (e.g., jsPsych, Cognition (www.cognition.run), JATOS (Lange et al., 2015), Gorilla, PsychoPy, Lab.js, LabVanced). While this diversity is positive in its own right, each tool faces a trade-off between flexibility (i.e., customizability) and ease of learning/use. Shiny stands out as an advantageous option for researchers familiarized with R, as it is highly *and* easily customizable (although specific features might require incorporating HTML/CSS/JavaScript code). Moreover, Shiny-app development can be readily integrated into the workflow of experimental psychologists that use R for data analysis without the need to switch back and forth between frameworks and/or programming languages.

Altogether, this study shows that a well-established RT-based cognitive effect can be robustly captured online, highlighting Shiny as a useful tool to develop replicable online experiments. We showed that online experiments are not only useful for survey-data collection but also for RT-based decision-making tasks, harnessing great power for experimental psychologists. This methodological contribution could pave the way for more massive and cost-efficient data collection protocols across cognitive science at large.

## Data Accessibility statement

The datasets generated and/or analyzed in this study (containing data from 19604 trials) are available in the OSF repository: https://osf.io/6gmzs.

## Code Accessibility

All code for the app and analyses are available in the OSF repository: https://osf.io/6gmzs. A demo version of the app is available in ShinyApps: https://2exp3.shinyapps.io/numbers/.

## Ethics and consent

This study was performed in line with the principles of the Declaration of Helsinki. Approval was granted by the Ethics Committee of Centro de Educación Médica e Investigaciones Clínica (protocol ID 435). The authors complied with APA ethical standards in the treatment of their sample.

Informed consent was obtained from all individual participants included in the study.

## Footnotes

Although in this paper the authors report the correlation coefficient between RT and logD, the regression coefficient is not provided. Since the data from this study is not publicly available, we scraped the data from the corresponding figure using WebPlotDigitizer (https://github.com/ankitrohatgi/WebPlotDigitizer) and employed the same statistical method described in the paper to obtain a rough estimate of the logD regression coefficient.

## References

[1] Anwyl-Irvine, A., Dalmaijer, E. S., Hodges, N., & Evershed, J. K. (2020). Realistic precision and accuracy of online experiment platforms, web browsers, and devices. Behavior Research Methods. DOI: 10.3758/s13428-020-01501-5 PMC8367876 33140376

[2] Bridges, D., Pitiot, A., MacAskill, M. R., & Peirce, J. W. (2020). The timing mega-study: Comparing a range of experiment generators, both lab-based and online. PeerJ, 8, e9414. DOI: 10.7717/peerj.9414 33005482 PMC7512138

[3] Brown, H. R., Zeidman, P., Smittenaar, P., Adams, R. A., McNab, F., Rutledge, R. B., & Dolan, R. J. (2014). Crowdsourcing for Cognitive Science – The Utility of Smartphones. PLOS ONE, 9(7), e100662. DOI: 10.1371/journal.pone.0100662 25025865 PMC4099129

[4] Bürkner, P.-C. (2017). brms: An R Package for Bayesian Multilevel Models Using Stan. Journal of Statistical Software, 80(1), 1–28. DOI: 10.18637/jss.v080.i01

[5] Carpenter, B., Gelman, A., Hoffman, M. D., Lee, D., Goodrich, B., Betancourt, M., Brubaker, M., Guo, J., Li, P., & Riddell, A. (2017). Stan: A Probabilistic Programming Language. Journal of Statistical Software, 76(1), 1–32. DOI: 10.18637/jss.v076.i01 PMC9788645 36568334

[6] Chang, W., Cheng, J., Allaire, J., Xie, Y., & McPherson, J. (2018). shiny: Web application framework for r, 2015. R Package Version, 1(0), 14.

[7] Cipora, K., Soltanlou, M., Reips, U.-D., & Nuerk, H.-C. (2019). The SNARC and MARC effects measured online: Large-scale assessment methods in flexible cognitive effects. Behavior Research Methods, 51(4), 1676–1692. DOI: 10.3758/s13428-019-01213-5 30805864

[8] Crump, M. J. C., McDonnell, J. V., & Gureckis, T. M. (2013). Evaluating Amazon’s Mechanical Turk as a Tool for Experimental Behavioral Research. PLOS ONE, 8(3), e57410. DOI: 10.1371/journal.pone.0057410 23516406 PMC3596391

[9] Dandurand, F., Shultz, T. R., & Onishi, K. H. (2008). Comparing online and lab methods in a problem-solving experiment. Behavior Research Methods, 40(2), 428–434. DOI: 10.3758/BRM.40.2.428 18522052

[10] de Leeuw, J. R. (2015). jsPsych: A JavaScript library for creating behavioral experiments in a Web browser. Behavior Research Methods, 47(1), 1–12. DOI: 10.3758/s13428-014-0458-y 24683129

[11] Dehaene, S. (1989). The psychophysics of numerical comparison: A reexamination of apparently incompatible data. Perception & Psychophysics, 45(6), 557–566. DOI: 10.3758/BF03208063 2740196

[12] Dehaene, S. (2011). The number sense: How the mind creates mathematics (Rev. and updated ed). Oxford University Press.

[13] Dehaene, S., Dupoux, E., & Mehler, J. (1990). Is numerical comparison digital? Analogical and symbolic effects in two-digit number comparison. Journal of Experimental Psychology: Human Perception and Performance, 16(3), 626–641. DOI: 10.1037/0096-1523.16.3.626 2144576

[14] Dufau, S., Duñabeitia, J. A., Moret-Tatay, C., McGonigal, A., Peeters, D., Alario, F.-X., Balota, D. A., Brysbaert, M., Carreiras, M., Ferrand, L., Ktori, M., Perea, M., Rastle, K., Sasburg, O., Yap, M. J., Ziegler, J. C., & Grainger, J. (2011). Smart Phone, Smart Science: How the Use of Smartphones Can Revolutionize Research in Cognitive Science. PLOS ONE, 6(9), e24974. DOI: 10.1371/journal.pone.0024974 21980370 PMC3182196

[15] Gabry, J. (2018). shinystan: Interactive visual and numerical diagnostics and posterior analysis for Bayesian models (Version 2.5.0) [Computer software].

[16] Garaizar, P., & Reips, U.-D. (2019). Best practices: Two Web-browser-based methods for stimulus presentation in behavioral experiments with high-resolution timing requirements. Behavior Research Methods, 51(3), 1441–1453. DOI: 10.3758/s13428-018-1126-4 30276629

[17] Gelman, A., Goodrich, B., Gabry, J., & Vehtari, A. (2019). R-squared for Bayesian Regression Models. The American Statistician, 73(3), 307–309. DOI: 10.1080/00031305.2018.1549100

[18] Gelman, A., Jakulin, A., Pittau, M. G., & Su, Y.-S. (2008). A weakly informative default prior distribution for logistic and other regression models. Annals of Applied Statistics, 2(4), 1360–1383. DOI: 10.1214/08-AOAS191

[19] Gelman, A., Vehtari, A., Simpson, D., Margossian, C. C., Carpenter, B., Yao, Y., Kennedy, L., Gabry, J., Bürkner, P.-C., & Modrák, M. (2020). Bayesian Workflow. ArXiv:2011.01808 [Stat]. http://arxiv.org/abs/2011.01808

[20] Germine, L., Nakayama, K., Duchaine, B. C., Chabris, C. F., Chatterjee, G., & Wilmer, J. B. (2012). Is the Web as good as the lab? Comparable performance from Web and lab in cognitive/perceptual experiments. Psychonomic Bulletin & Review, 19(5), 847–857. DOI: 10.3758/s13423-012-0296-9 22829343

[21] Gökaydin, D., Brugger, P., & Loetscher, T. (2018). Sequential Effects in SNARC. Scientific Reports, 8(1), 10996. DOI: 10.1038/s41598-018-29337-2 30030492 PMC6054671

[22] Gosling, S. D., Vazire, S., Srivastava, S., & John, O. P. (2004). Should We Trust Web-Based Studies? A Comparative Analysis of Six Preconceptions About Internet Questionnaires. American Psychologist, 59(2), 93–104. DOI: 10.1037/0003-066X.59.2.93 14992636

[23] Grootswagers, T. (2020). A primer on running human behavioural experiments online. Behavior Research Methods, 52(6), 2283–2286. DOI: 10.3758/s13428-020-01395-3 32291730

[24] Hinrichs, J. V., Yurko, D. S., & Hu, J. (1981). Two-digit number comparison: Use of place information. Journal of Experimental Psychology: Human Perception and Performance, 7(4), 890–901. DOI: 10.1037/0096-1523.7.4.890

[25] Holden, J., Francisco, E., Lensch, R., Tommerdahl, A., Kirsch, B., Zai, L., Dennis, R., & Tommerdahl, M. (2019). Accuracy of different modalities of reaction time testing: Implications for online cognitive assessment tools. BioRxiv, 726364. DOI: 10.1101/726364

[26] Hosmer Jr, D. W., Lemeshow, S., & Sturdivant, R. X. (2013). Applied Logistic Regression. John Wiley & Sons. DOI: 10.1002/9781118548387

[27] Huber, S., Nuerk, H.-C., Reips, U.-D., & Soltanlou, M. (2019). Individual differences influence two-digit number processing, but not their analog magnitude processing: A large-scale online study. Psychological Research, 83(7), 1444–1464. DOI: 10.1007/s00426-017-0964-5 29275433

[28] Kaufman, A. R. (2020). Implementing novel, flexible, and powerful survey designs in R Shiny. PLOS ONE, 15(4), e0232424. DOI: 10.1371/journal.pone.0232424 32353057 PMC7192460

[29] Kochari, A. R. (2019). Conducting Web-Based Experiments for Numerical Cognition Research. Journal of Cognition, 2(1). DOI: 10.5334/joc.85 PMC6753310 31576378

[30] Krajcsi, A., & Kojouharova, P. (2017). Symbolic Numerical Distance Effect Does Not Reflect the Difference between Numbers. Frontiers in Psychology, 8. DOI: 10.3389/fpsyg.2017.02013 PMC5715324 29250002

[31] Krantz, J. H., & Dalal, R. (2000). Validity of Web-based psychological research. In Psychological experiments on the Internet (pp. 35–60). Academic Press. DOI: 10.1016/B978-012099980-4/50003-4

[32] Lange, K., Kühn, S., & Filevich, E. (2015). “Just Another Tool for Online Studies” (JATOS): An Easy Solution for Setup and Management of Web Servers Supporting Online Studies. PLOS ONE, 10(6), e0130834. DOI: 10.1371/journal.pone.0130834 26114751 PMC4482716

[33] Lieberoth, A. (2015). Shallow Gamification: Testing Psychological Effects of Framing an Activity as a Game. Games and Culture, 10(3), 229–248. DOI: 10.1177/1555412014559978

[34] Lumsden, J., Edwards, E. A., Lawrence, N. S., Coyle, D., & Munafò, M. R. (2016). Gamification of Cognitive Assessment and Cognitive Training: A Systematic Review of Applications and Efficacy. JMIR Serious Games, 4(2), e5888. DOI: 10.2196/games.5888 PMC4967181 27421244

[35] Moyer, R. S., & Landauer, T. K. (1967). Time required for judgements of numerical inequality. Nature, 215(5109), 1519–1520. DOI: 10.1038/2151519a0 6052760

[36] Moyer, R. S., & Landauer, T. K. (1973). Determinants of reaction time for digit inequality judgments. Bulletin of the Psychonomic Society, 1(3), 167–168. DOI: 10.3758/BF03334328

[37] Munafò, M. R., Nosek, B. A., Bishop, D. V. M., Button, K. S., Chambers, C. D., Percie du Sert, N., Simonsohn, U., Wagenmakers, E.-J., Ware, J. J., & Ioannidis, J. P. A. (2017). A manifesto for reproducible science. Nature Human Behaviour, 1(1), 1–9. DOI: 10.1038/s41562-016-0021 PMC7610724 33954258

[38] Nuerk, H.-C., Weger, U., & Willmes, K. (2001). Decade breaks in the mental number line? Putting the tens and units back in different bins. Cognition, 82(1), B25–B33. DOI: 10.1016/S0010-0277(01)00142-1 11672709

[39] Plant, R. R. (2016). A reminder on millisecond timing accuracy and potential replication failure in computer-based psychology experiments: An open letter. Behavior Research Methods, 48(1), 408–411. DOI: 10.3758/s13428-015-0577-0 25761394

[40] Pronk, T., Wiers, R. W., Molenkamp, B., & Murre, J. (2020). Mental chronometry in the pocket? Timing accuracy of web applications on touchscreen and keyboard devices. Behavior Research Methods, 52(3), 1371–1382. DOI: 10.3758/s13428-019-01321-2 31823223 PMC7280355

[41] Ratcliff, R., Thapar, A., Gomez, P., & McKoon, G. (2004). A Diffusion Model Analysis of the Effects of Aging in the Lexical-Decision Task. Psychology and Aging, 19(2), 278–289. DOI: 10.1037/0882-7974.19.2.278 15222821 PMC1360155

[42] Ratcliff, R., Thapar, A., & McKoon, G. (2010). Individual differences, aging, and IQ in two-choice tasks. Cognitive Psychology, 60(3), 127–157. DOI: 10.1016/j.cogpsych.2009.09.001 19962693 PMC2835850

[43] Reimers, S., & Stewart, N. (2008). Using Adobe Flash Lite on mobile phones for psychological research: Reaction time measurement reliability and interdevice variability. Behavior Research Methods, 40(4), 1170–1176. DOI: 10.3758/BRM.40.4.1170 19001409

[44] Reips, U.-D. (2002). Standards for Internet-Based Experimenting. Experimental Psychology, 49(4), 243–256. DOI: 10.1026//1618-3169.49.4.243 12455331

[45] Semmelmann, K., & Weigelt, S. (2017). Online psychophysics: Reaction time effects in cognitive experiments. Behavior Research Methods, 49(4), 1241–1260. DOI: 10.3758/s13428-016-0783-4 27496171

[46] Silver, L., Smith, A., Johnson, C., Taylor, K., Jiang, J., Anderson, M., & Rainie, L. (2019). Mobile connectivity in emerging economies. Pew Research Center, 7.

[47] Simcox, T., & Fiez, J. A. (2014). Collecting Response Times using Amazon Mechanical Turk and Adobe Flash. Behavior Research Methods, 46(1), 95–111. DOI: 10.3758/s13428-013-0345-y 23670340 PMC5283577

[48] Steiner, M., Phillips, N., & Trutmann, K. (2020). ShinyPsych: An easy way to program psychology experiments using Shiny.

[49] Verguts, T., & De Moor, W. (2005). Two-digit Comparison. Experimental Psychology, 52(3), 195–200. DOI: 10.1027/1618-3169.52.3.195 16076067

[50] Wagenmakers, E.-J., & Brown, S. (2007). On the linear relation between the mean and the standard deviation of a response time distribution. Psychological Review, 114(3), 830–841. DOI: 10.1037/0033-295X.114.3.830 17638508

[51] Wiley, K., Vedress, S., & Mandryk, R. L. (2020). How Points and Theme Affect Performance and Experience in a Gamified Cognitive Task. In Proceedings of the 2020 CHI Conference on Human Factors in Computing Systems (pp. 1–15). Association for Computing Machinery. DOI: 10.1145/3313831.3376697

[52] Zimmerman, F., Shalom, D., Gonzalez, P. A., Garrido, J. M., Heduan, F. A., Dehaene, S., Sigman, M., & Rieznik, A. (2016). Arithmetic on Your Phone: A Large Scale Investigation of Simple Additions and Multiplications. PLOS ONE, 11(12), e0168431. DOI: 10.1371/journal.pone.0168431 28033357 PMC5199052

